# Effects of Chenpi Jiaosu on serum metabolites and intestinal microflora in a dyslipidemia population: a randomized controlled pilot trial

**DOI:** 10.3389/fendo.2025.1552117

**Published:** 2025-03-28

**Authors:** Fei Tan, Yuying Zheng, Chengcheng Wang, Jiaying Huang, Xin Liu, Weiwei Su, Xinyan Chen, Zhimin Yang

**Affiliations:** ^1^ State Key Laboratory of Dampness Syndrome of Chinese Medicine, The Second Affiliated Hospital of Guangzhou University of Chinese Medicine, Guangzhou, China; ^2^ Guangdong Engineering Research Center of Commercialization of Medical Institution Preparations and Traditional Chinese Medicines, Engineering Technology Research Center of Commercialization of Linnan Special Medical Institution Preparations, Experimental Center, The First Affiliated Hospital of Guangzhou University of Chinese Medicine, Guangzhou, China; ^3^ Guangdong Clinical Research Academy of Chinese Medicine, The First Affiliated Hospital of Guangzhou University of Chinese Medicine, Guangzhou, China; ^4^ Guangdong Engineering Research Center of Commercialization of Medical Institution Preparations and Traditional Chinese Medicines, Guangzhou, China; ^5^ Guangdong Engineering Technology Research Center of Commercialization of Linnan Special Medical Institution Preparations, Guangzhou, China; ^6^ The TCM Department of Longyuan Daguan Community Health Service Center, Shenzhen Longgang Orthopaedics Hospital, Shenzhen, China; ^7^ Production department, Guangzhou Baiyunshan Guanghua Pharmaceutical co, LTD, Guangzhou, China; ^8^ School of Life Sciences, Sun Yat-sen University, Guangzhou, China

**Keywords:** Chenpi Jiaosu, dyslipidemia, metabolites, intestinal microflora, healthy food

## Abstract

**Introduction:**

Dyslipidemia is a critical risk factor for atherosclerosis and cardiovascular/cerebrovascular events, necessitating effective long-term management. However, conventional lipid-lowering drugs such as statins and fibrates are limited by adverse effects, including hepatotoxicity and myopathy, which restrict their prolonged use. Traditional Chinese medicine (TCM) and natural health products offer potential alternatives with multi-target mechanisms and improved safety profiles. Tangerine Peel Enzyme Drink (CPJS), a fermented health product derived from tangerine peel, has demonstrated lipid-modulating properties. This study aimed to evaluate the efficacy and safety of CPJS in improving dyslipidemia and explore its underlying metabolic and microbiological mechanisms.

**Methods:**

A randomized, double-blind, parallel-controlled clinical trial was conducted with 72 participants (55 completers). Participants were divided into CPJS and control groups, receiving an 8-week intervention. Primary outcomes included changes in body weight and serum triglycerides (TG), while safety was assessed via liver/kidney function, creatine kinase, blood, and urine tests. Serum metabolomics (93 differential metabolites identified) and intestinal microbiota analysis were performed to elucidate metabolic pathways and microbial shifts. KEGG enrichment analysis mapped metabolites to biological pathways, such as lipid and amino acid metabolism.

**Results:**

The CPJS group exhibited significant reductions in body weight and TG levels post-intervention (p < 0.05), with no adverse effects observed in safety biomarkers. Metabolomic profiling revealed alterations in fatty acyl, glycerophospholipid, and organic acid metabolites, indicating CPJS modulates lipid metabolism and energy homeostasis. KEGG analysis linked these changes to pathways including triglyceride degradation and amino acid metabolism. Additionally, CPJS increased specific gut microbial taxa associated with lipid regulation, suggesting a microbiome-mediated mechanism.

**Discussion:**

CPJS demonstrates efficacy in improving dyslipidemia through dual mechanisms: direct modulation of triglyceride metabolism and indirect regulation via gut microbiota. Its safety profile aligns with findings from natural products like Cyclocarya paliurus and tempeh, which mitigate lipid abnormalities without hepatotoxicity. The multi-target action of CPJS mirrors TCM principles, where compounds like quercetin and flavonoids in CPJS may synergistically inhibit cholesterol synthesis and enhance lipid clearance. However, further research is needed to isolate active components and validate microbial contributions. Compared to synthetic drugs, CPJS offers a safer adjunct therapy, addressing limitations of current pharmacotherapies. Future studies should explore dose-response relationships and long-term outcomes in diverse populations.

## Introduction

Cardiovascular diseases (CVDs) are the leading cause of global mortality and a significant contributor to disability (1). China and India bear the highest burden of CVD, accounting for 40% of deaths in the Chinese population (2). Extensive evidence indicates the efficacy of cholesterol-lowering therapies in mitigating the risk of CVD, prompting recent advocacy for the prevention and control of dyslipidemia as a crucial risk factor (3–5). Data from the China Nutrition Survey in 2012 revealed a prevalence of dyslipidemia at 40.4% (6, 7). Consequently, early diagnosis, treatment, and control of dyslipidemia, along with preventing or delaying its progression represent vital measure to decrease the incidence of CVD.

Despite the availability of various lipid-lowering drugs with diverse mechanisms of action in the market, their use is often limited due to concerns regarding side effects and insufficient efficacy (8). Many herbs have demonstrated safety and efficacy in treating dyslipidemia, with tangerine peel (“Chenpi” in Chinese) being a commonly used herb. Prior research has indicated that Chenpi can alleviate cough, bloating, and obesity (9–12). Classified as a homology of medicine and food, plant-based foods abundant in phytochemicals are increasingly gaining popularity and proving effective in disease management.

Chenpi is the dried and mature peel of *Citrus reticulata* Blanco and its cultivated varieties within the Rutaceae family. The primary components of Chenpi include flavonoids (such as hesperidin and naringin) and polymethyloxyl flavonoids (like nobiletin, sweet orange flavone, and hesperetin), as well as volatile oil components such as 2-methylamino-benzoate, D-limonene, and γ-terpinene (13, 14). Hesperidin exhibits antioxidant and anti-inflammatory properties (10), protects the cardiovascular system (12, 15), safeguards the nervous system (15), and enhances blood glucose and lipid metabolism (16), potentially reducing oxidative stress by 30% and the risk of cardiovascular disease by 25% (17). Nobiletin and other polymethoxy flavonoids possess modulatory effects, preventing dyslipidemia, protecting the myocardium and blood vessels, inhibiting platelet aggregation, and regulating the nerve center (18). Crude fiber also exhibits a notable ameliorative effect on elevated lipid profile (19). These compounds have demonstrated excellent performance in the prevention and treatment of cardiovascular diseases and the improvement of prognosis. Additionally, the technology employed in the use of Chenpi holds promising potential in the treatment of dyslipidemia.

Enzyme drinks, referred to as Jiaosu in China, are products containing specific biologically active ingredients obtained by microbial fermentation using animals, plants, fungi, etc., with or without auxiliary materials (20). The probiotics of Jiaosu can hydrolyze macromolecular substances, produce enzymes, and enhance the content of phenolic compounds, vitamins, minerals, and other metabolites (21). Given the intricate production of traditional Chinese medicine decoctions, we developed Jiaosu with Chenpi as the primary ingredient, supplemented with other medicinal and food homologous herbs such as ginseng, lycium, hawthorn, ginger, and Poria cocos. These ingredients are fermented together, rendering the product portable and easy to preserve. Tangerine Peel Enzyme Drink (“Chenpi Jiaosu” in Chinese, abbreviated as CPJS) is currently marketed as a health food in China. CPJS contains a variety of bioactive compounds; it exhibits blood lipid lowering, anticancer, and immunity-enhancing properties (22). Recent studies have revealed that Jiaosu possesses numerous beneficial functions, including antioxidant activity, liver detoxification, intestinal lubrication, and bowel evacuation (23).

To assess the potential of CPJS in treating dyslipidemia, we conducted a randomized controlled trial (RCT) in a dyslipidemia population. Our hypothesis posits that oral administration of CPJS will effectively reduce blood lipid level in adults with dyslipidemia. The primary endpoints focused on adjusting lipid biochemical parameters, including TC, TG, HDL-C, and LDL-C levels. Secondary endpoints encompassed the levels of BMI, GLU, UA, and CRP and other lipid components. Serum and fecal samples were collected from participants before and after CPJS intervention. Metabolomics and intestinal microbial flora were detected and analyzed, respectively. The results indicated that CPJS influenced energy metabolism, lipid metabolism, amino acid metabolism, and other pathways, while it increased the abundance of related intestinal microorganisms, including probiotics.

## Method

### Participants, sample collection, and study design

A prospective, single-center, randomized, placebo-controlled, parallel-group clinical trial was conducted to evaluate the safety and efficacy of CPJS in individuals aged 18–70 years with dyslipidemia. The participants were diagnosed based on the criteria outlined in the *Chinese Guidelines for the Prevention and Treatment of Dyslipidemia in Adults (2016 Revision)*, with a cardiovascular risk level classified as low or intermediate risk (24, 25).

The study was carried out at the Guangdong Provincial Hospital of Chinese Medicine (Guangzhou, China) from June 2020 and December 2020. Ethical approval was obtained from the hospital’s Ethics Committee (BF 2019-146-01), and the trial was registered with the Chinese Clinical Trial Registry (Identifier ChiTR2000033062, https://www.chictr.org.cn/). The study was performed according to the Declaration of Helsinki, and written informed consent was obtained from the participants prior to their enrollment.

Serum and fecal samples were collected from the participants before and after the treatment period. The serum samples were obtained following a comprehensive health examination at the Guangdong Provincial Hospital of Chinese Medical. Blood biochemistry measurements were conducted at the same hospital. Approximately 2 mL of fasting venous blood was drawn from the participants’ arms between 8 AM to 9 AM, prior to breakfast. Female participants were excluded if they were menstruating at the time of blood collection. The blood samples were solidified for 1 to 2 h (without anticoagulant) and then centrifuged after being stored statically at 4°C overnight. The insoluble substance was discarded, and the serum was transferred to clean test tubes. The obtained samples were stored at −80°C until further analysis.

CPJS, the investigational product, is a functional food formulation produced by the co-fermentation of several medicinal and edible homologous materials, including Xinhui citrus fruit flesh, Xinhui tangerine peel, lycium, ginseng, hawthorn, ginger, and Poria cocos (26) The CPJS used in this study is currently available in the market, and the quality control standard of CPJS is detailed in **Supplementary Data Sheet 1**. The placebo used in the study consisted of a 5% concentration of CPJS.

The primary endpoints of the study were changes in blood lipid levels, including total cholesterol (TC), triglyceride (TG), high-density lipoprotein cholesterol (HDL-C), and low-density lipoprotein cholesterol (LDL-C). The secondary endpoints included body parameters related to lipid metabolism, such as body mass index (BMI), waistline circumference, and hip circumference.

### Randomization

The participants were randomized into two groups in equal proportions using the SAS statistical software PROC PLAN procedure to generate a random sequence for the allocation of participants to the study interventions: CPJS (treatment) or placebo. A project researcher prepared the intervention products and provided them to the participants, who were blinded to the product, according to a computer-generated randomization list created by an independent biostatistician not involved in the study. The CPJS and placebo were identical in appearance to ensure blinding. Code envelopes for emergency unblinding were securely maintained by both the sponsor and the investigator. The participants were instructed to take the study products twice daily (at 10 AM and 4 PM) for a total of 56 days.

### Power calculation

CPJS is a healthy food, and no previous data of similar studies have been seen before. This was an exploratory study based on the number of patients available who fit the inclusion and exclusion criteria. To adjust for a 20% drop-out rate, we decided to enroll 72 volunteers into the trial.

### Procedures

All participants underwent a baseline blood lipid parameter evaluation, including blood biochemical and body shape measurements, by physicians who were masked to the group assignments. The participants completed the blood draws in a hospital laboratory and completed a baseline information collection and assessment in a quiet room with physicians who were uniformly trained on administering the secondary outcome measures and who were masked to the group assignments. Meetings were held with the evaluators at monthly intervals to maintain consistency with evaluation performance and review protocols. The participants were paid ¥800 after completing the treatment and pre- and post-testing sessions.

### UHPLC-Q-TOF-MS/MS analysis

All serum samples were stored in aliquots at -80°C and thawed on ice prior to analysis. The serum (100 μL) of each sample was mixed with methanol/acetonitrile (1:1, v/v) (200 μL) liquid containing myristic acid-D27 (98%, Cambridge Isotope Laboratories Inc., Andover, USA) (20 μg/mL as internal standard) and vortexed for 30 s. After 1 h of incubating at 20°C in order to precipitate proteins, all mixtures were centrifuged at 15,000 *g* for 20 min at 4°C, and the supernatant was collected (200 μL). The quality control (QC) sample was a 5-μL aliquot of each test sample solution that was mixed for method validation.

The UPLC-QTOF-MS analysis was performed by ultra-fast liquid chromatography (Shimadzu Corp., Kyoto, Japan) coupled with quadrupole/time-of-flight mass spectrometry (Triple TOF 5600 plus, AB SCIEX, Foster City, CA, USA) through an electrospray ionization (ESI) interface. ACQUITY UPLC HSS C_18_ (100 mm × 2.1 mm, 1.8 μL, Waters Co., Milford, MA, USA) was used for chromatographic separation. The column temperature was set at 50°C, and the room temperature was set at 16°C. The mobile phase consisted of eluent A (0.1% formic acid in aqueous solution) and eluent B (0.1% formic acid in water solution). The gradient elution program was set up as follows: 0–1.5 min, 10% B; 1.5–13 min, 1%–99% A; 13–16.5 min, 90% A; 16.5–17 min, 99%–1% A; 17–20 min, 1% A with the flow rate at 0.3 mL/min.

MS/MS identification was conducted using an electrospray ionization (ESI) source with the following parameters. The ion spray voltage was 5,500 V in positive ion mode while −4,500 V in negative ion mode. The mass range was from 50 to 1,500 Da. The ion source gas 1 and gas 2 were both 55 psi, and the curtain gas was set as 35 psi. The ion source temperature was maintained at 550°C. The declustering potential was 80 V. The collision energy and its spread were set as 30 and 15 V, respectively. Nitrogen was used as the nebulizer and auxiliary gas. Data acquisition was carried out using Analyst^®^ 1.2 software (AB Sciex, Foster City, CA, USA) in the information-dependent acquisition mode.

### Metabolomics study

The raw data from the UPLC-Q/TOF-MS/MS system were processed with One-MAP online platform (http://www.5omics.com/) for format conversion, mass spectrometry extraction, and qualitative identification analysis. In addition, the obtained peak table is imported into MetaboAnalyst (https://www.metaboanalyst.ca/) for peak missing value filling and normalization process. The processed data were entered into SIMCA-P software (Version 13) for orthogonal projection to latent structures discriminant analysis (OPLS-DA). S-plots based on OPLS-DA predictions could demonstrate potential biomarkers which significantly contribute to metabolic differences. Metabolites with the variable importance in the projection (VIP) values above 1.0 and *p*-value below 0.05 were considered significantly different. Pathway enrichment analysis was performed using MetaboAnalyst and KEGG (http://www.genome.jp/kegg).

### Gut microbiota analysis using 16S rRNA gene sequencing

Advanced third-generation sequencing technology was utilized for gut microbiome analysis. Bacterial DNA was extracted using the PowerSoil DNA Isolation kit (MO BIO Laboratories, USA). The full-length 16S rRNA gene was PCR-amplified using the bacterial primers 27F (5′-AGRGTTYGATYMTGGCTCAG-3′) and 1492R (5′-RGYTACCTTGTTACGACTT-3′). After PCR amplification, sequencing was conducted on the PacBio platform by Biomarker Technologies Co. Ltd. (Beijing, China).

Circular consensus sequencing (CCS) reads were obtained using SMRT Link v8.0 with parameters (minPasses ≥5, minPredictedAccuracy ≥0.9). These Optimized-CCS reads were clustered into operational taxonomic units (OTUs) based on 97% sequence similarity using USEARCH v10.0. The sequences were annotated using the SILVA bacterial 16S rRNA database. The detected communities were identified and annotated at different taxonomic levels.

The alpha-diversity of the gut microbiota was analyzed using ACE, Chao1, Simpson, and Shannon indices. The beta-diversity was evaluated using principal coordinates analysis (PCoA) based on Binary–Jaccard distances. Linear discriminant analysis (LDA) effect size (LEfSe) was employed to identify statistically significant biomarkers between the placebo and CPJS groups. At the species level, taxa with an LDA score >2 and *p*-value <0.05 (Wilcoxon rank-sum test) were considered differentially abundant. All processes were executed on the BMKCloud platform (www.biocloud.net).

### Statistical analysis

Data normality was assessed by using the Kolmogorov–Smirnov method. Comparisons between the two groups were performed by using *t*-test. Student’s *t*-test was used to calculate the *p*-value with homogeneity of variance; conversely, Welch’s *t*-test was used to calculate the *p*-value. Mann–Whitney–Wilcoxon test was used to analyze non-normal data. Clinical data were analyzed descriptively as frequencies, percentages, means and standard deviations, and 95% confidence intervals. All statistical analyses about the identification of distinct metabolites were completed with R (v4.0.2) basic statistical packages. All statistical significance values were accepted at *p*-value <0.05.

## Results

**Figure 1:**
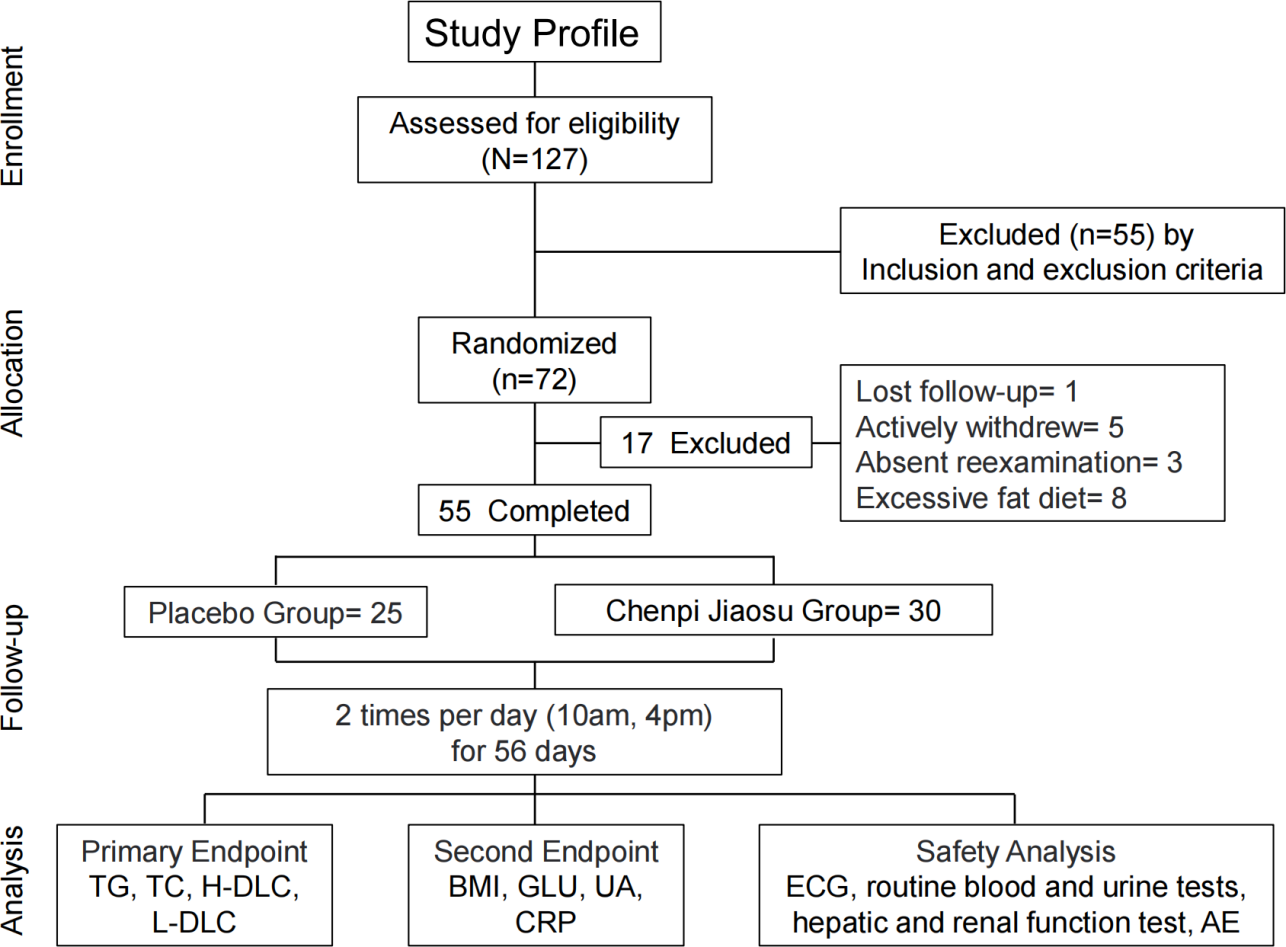
Flow diagram of the randomized controlled pilot trial. TG, triglyceride; TC, total cholesterol; HDL-C, high-density lipoprotein cholesterol; LDL-C, low-density lipoprotein cholesterol; BMI, body mass index; GLU, glucose; UA, uric acid; CRP, C-reactive protein; ECG, electrocardiogram; AE, adverse event.

A total of 127 volunteers were screened for this study. Of these, 55 were excluded based on the exclusion criteria, and 72 met all of the eligibility criteria and were randomized. During the study, one participant was lost to follow-up, five withdrew early due to personal reasons, and three completed only the partial evaluations. No adverse effects were reported in either treatment group. Additionally, eight participants were excluded from analysis due to excessive fat intake during the study period. The study flow chart is illustrated in **Figure 1**. Consequently, 55 participants (76% of the randomized cohort) were included in the final analysis, with 30 in the treatment group and 25 in the placebo group. No significant differences were observed between the two groups in terms of age, gender, weight, waist circumference, or hip circumference. The demographic and body parameters for both groups are summarized in **Table 1**.

**Table 1:** Participant demographics.

Characteristic	Placebo Group (n=25). *M (SD)* or n	CPJS Group (n=30). *M (SD) *or n
Age, yr	54.74 (11.00)	52.50 (11.90)
Gender		
Male	10	10
Female	20	15
Body weight, kg	61.71 (9.57)	66.69 (11.12)
Waistline, cm	67.23 (8.41)	66.84 (6.79)
Hip circumference, cm	77.77 (10.75)	77.04 (10.12)
BMI, kg/m2	23.54 (3.10)	24.84 (2.70)

*M*, Mean; *SD*, Standard deviation; CPJS, Chenpi Jiaosu; BMI, Body mass index.

### Safety analysis

Safety analysis was conducted for the 55 participants. No adverse reactions were observed during the administration of CPJS or placebo. Evaluations of electrocardiogram (ECG), liver function, kidney function, hematological parameters, and urological function were in normal ranges both before and after the treatment period (**Table 2**).

**Table 2:** Safety indexes of participants before and after treatment.

Parameter Measure	Timepoints	Placebo Group (n=25). *M (SD)*	CPJS Group (n=30). *M* (SD)	*P* value (Intra-group)		*P* value (Inter-group comparison of difference)
				Placebo Group	CPJS Group	
ALT, U/L	Baseline (0w)	22.33 (12.98)	24.16 (16.25)	0.588	0.608	0.959
	Posttreatment (8w)	21.25 (9.90)	22.92 (10.38)			
AST, U/L	Baseline (0w)	21.57 (7.20)	21.48 (5.01)	0.480	0.521	0.986
	Posttreatment (8w)	22.30 (7.62)	22.24 (7.55)			
CR, umol/L	Baseline (0w)	74.27 (17.62)	71.40 (14.27)	0.000**	0.000**	0.428
	Posttreatment (8w)	67.26 (14.84)	63.00 (13.53)			
BUN, mmol/L	Baseline (0w)	4.89 (1.09)	4.83 (1.23)	0.318	0.309	0.470
	Posttreatment (8w)	5.04 (1.16)	4.64 (1.41)			

ALT, Alanine aminotransferase; AST, Aspartate aminotransferase; CR, Creatinine; BUN, Blood Urea Nitrogen. Intra-group comparison after treatment, p**≤0.01.

### Clinical efficacy of oral administration of Chenpi Jiaosu

**Figure 2:**
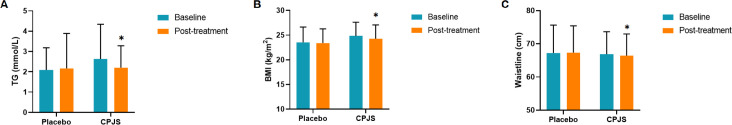
**(A–C)** Measure scores of primary and secondary outcomes with significant improvement. TG, triglyceride; BMI, body mass index. *p ≤ 0.05.

At enrollment, there were no significant differences between the two groups in terms of gender, age, blood lipid levels, and BMI. Among the 55 participants evaluated, after 8 weeks of intervention, no significant change in blood lipid levels was observed in the placebo group before and after the treatment. However, in the treatment group, there was a significant decrease in the average TG levels by 0.43 mmol/(**Figure 2**). Other blood lipid indices, including TC, HDL-C, and LDC-L, as well as metabolic level indicators such as GLU and UA showed minimal changes in both groups before and after the intervention (**Supplementary Figure 1**). We also assessed the effect of the intervention on body mass indicator. The mean BMI in the treatment group decreased significantly by 0.56 kg/m^2^ (from 24.84 to 24.28 kg/m^2^), while the placebo group showed a smaller reduction from 23.54 to 23.41 kg/m^2^, which was not statistically significant. The difference in BMI changes between the two groups was statistically significant (**Figure 2**) Similarly, hip circumference in the treatment group decreased significantly by 0.56 cm, from 77.04 to 76.48 cm. In contrast, the placebo group showed a smaller reduction from 77.77 to 77.53 cm, which was not statistically significant. The difference in hip circumference changes between the two groups was also statistically significant (**Figure 2**). No significant changes were observed in waistline, systolic blood pressure, or diastolic pressure after the intervention (**Supplementary Table 1**).

### Multivariate analysis of metabolomics data

**Figure 3:**
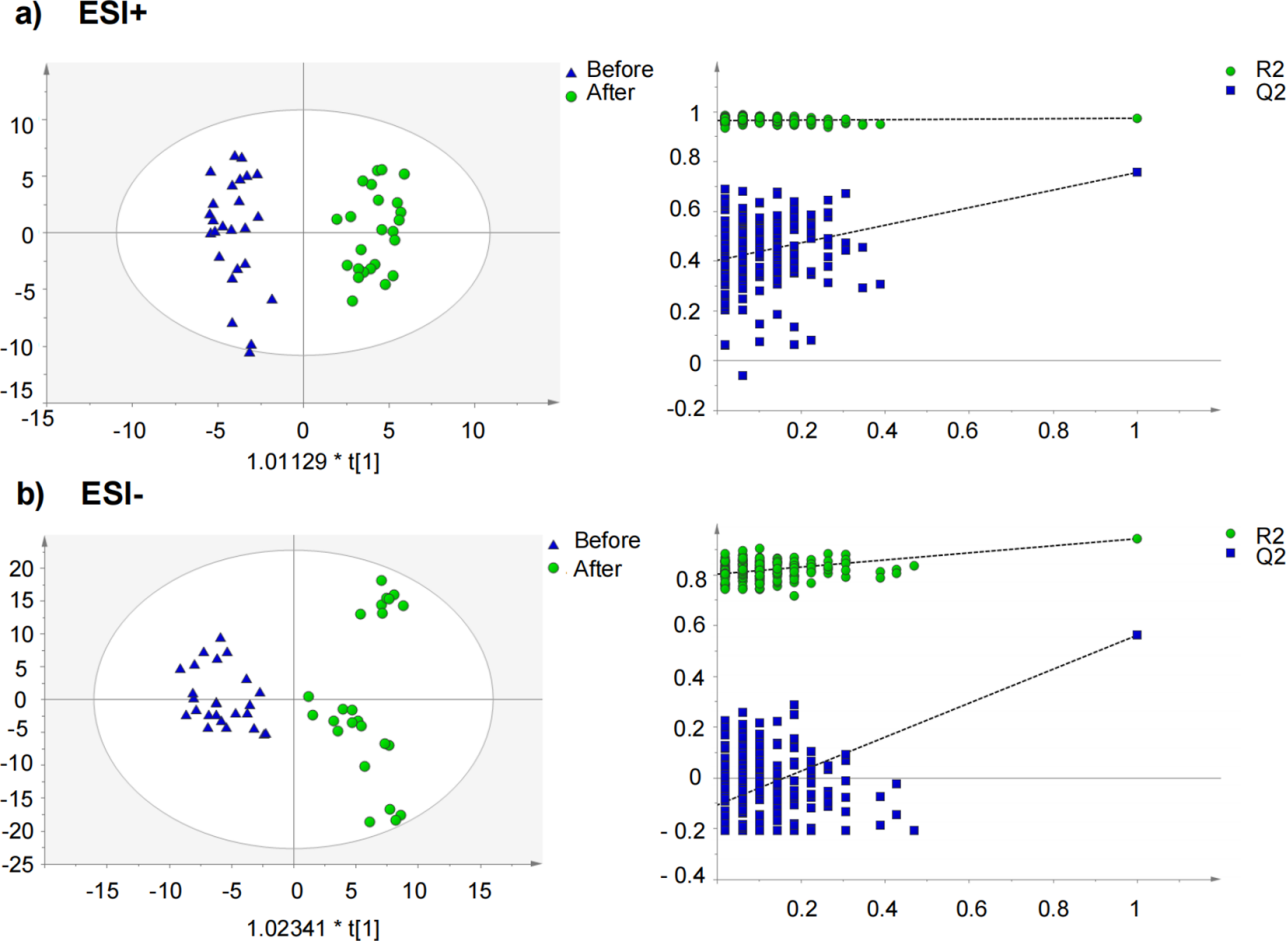
Orthogonal partial least squares discriminant analysis (OPLS-DA) score plot and permutation test for the CPJS group before and after treatment in **(a)** negative mode and in **(b)** positive mode.

To further elucidate the differential metabolites between the two groups, non-targeted metabolomics was conducted to serum samples using UPLC-QTOF-MS/MS. Orthogonal Projections to Latent Structures Discriminant Analysis (OPLS-DA), a supervised method of pattern recognition, was employed to visualize and depict the general metabolic variations between the CPJS group and the placebo group. As shown in **Figure 3**, each sample is represented as a single spot in the score plots, and the two groups are clearly separated in ESI+ and ESI modes. These findings suggest that oral administration of CPJS can alter the serum metabolic profile of the participants with dyslipidemia and regulate the levels of certain metabolites. Permutation tests (*n* = 200) were used to validate the OPLS-DA model. The results indicate that the model is effective, exhibits high stability and predictive ability, and is free from overfitting.

### Identification of the differential metabolites and metabolic pathways in serum

Potentially differential metabolites were selected based on their variable importance for the projection (VIP) scores extracted from the OPLS-DA models mentioned above. A total of 26 robust endogenous metabolites in serum were identified as potential biomarkers. These metabolites were marked in red in the S-plots and selected based on the criteria of VIP >1.0, *p <*0.05, and confirmation through mass spectrometry comparison. To further validate the identities of these metabolites, we compared their spectra with referenced spectra from HMDB, Metelin, and Pubchem. Detailed information of fragments used for identification is provided in **Supplementary Table 2**. Overall, a total of 93 different metabolites were identified, primarily belonging to the categories of fatty acyl, glycerophospholipid, and organic acid.

**Figure 4:**
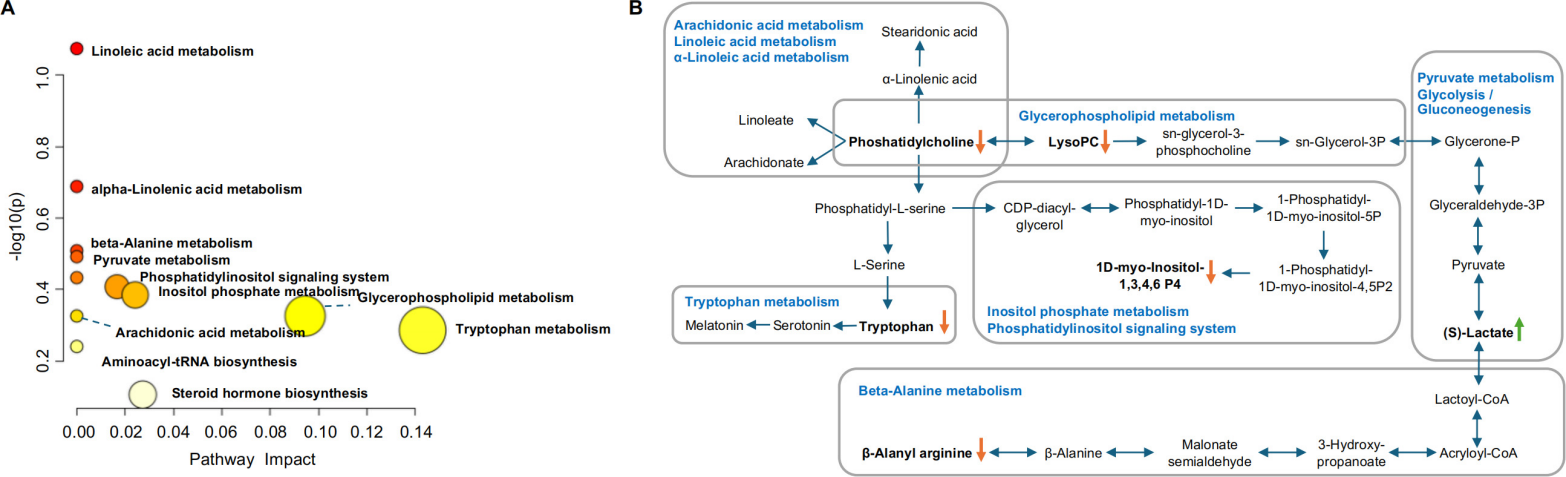
Identification of the metabolic pathways in serum samples. **(A)** Metabolic pathway analysis results based on the 12 regulated serum metabolites after CPJS treatment. **(B)** Summaries of metabolic pathways. The orange downward arrow indicates downregulation, while the green upward arrow signifies upregulation.

The metabolic pathway analysis using MetaboAnalyst 4.0 based on KEGG database (www.keggjp) revealed that 12 pathological processes were associated with CPJS intervention, including linoleic acid metabolism, α-linolenic acid metabolism, beta-alanine metabolism, pyruvate metabolism, glycolysis/gluconeogenesis, phosphatidylinositol signaling system, inositol phosphate metabolism, arachidonic acid metabolism, glycerophospholipid metabolism and tryptophan metabolism, aminoacyl-tRNA biosynthesis, and steroid hormone biosynthesis (**Figure 4**). The important differential metabolites are summarized in **Figure 4**, and we found that glycerophospholipid metabolism plays an important role in the protection of CPJS against dyslipidemia. The impact value represents the importance of the metabolic pathways, the -log10(*P*) value represents the difference in the metabolic pathway, and the size of the circle is positively correlated with the two parameters mentioned above. The identified metabolic pathways are summarized in **Supplementary Table 3**.

### Gut microbiota modulatory effects of Chenpi Jiaosu

After 56 days of dietary intervention, feces samples were collected for an analysis of the gut microbiota community in the participants. A total of 854,945 high‐quality sequences of the V1–V9 region of 16S rRNA were obtained from 112 feces samples. The average sequence number was 4,011. Based on 97% similarity level, all of the effective reads were clustered into OTUs. At the phylum level, the most abundant bacteria were Bacteroidetes (48.7%), Firmicutes (36.1%), Proteobacteria (9.5%), Fusobacteriota (4.3%), Actinobacteriota (0.6%), and Verrucomicrobiota (0.3%).

**Figure 5:**
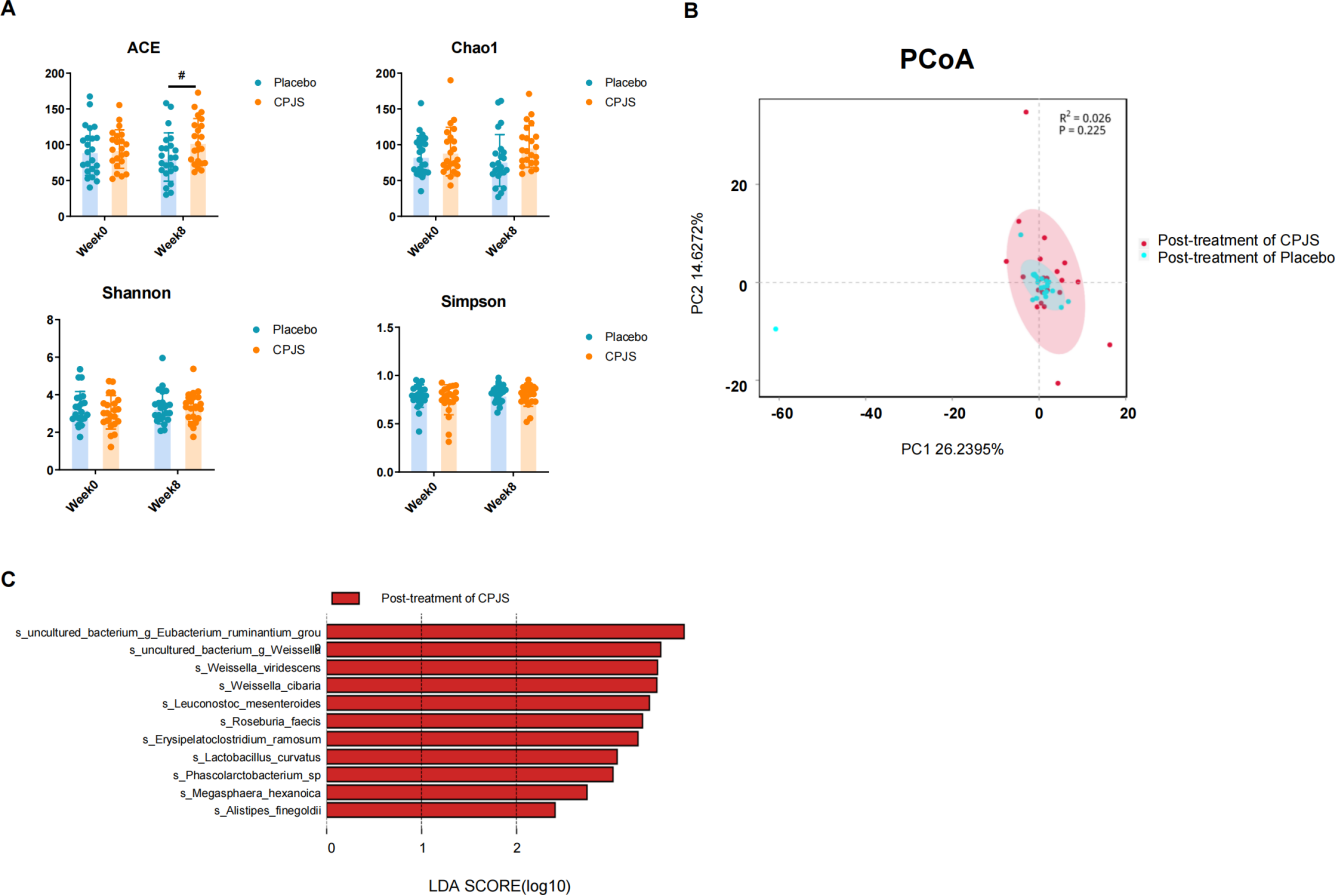
Gut microbiota modulatory effects after oral administration of Chenpi Jiaosu. **(A)** Diversity index, including ACE (a), Chao1 (b), Shannon (c), and Simpson index (d), of gut microbiota in participants of the placebo group (*n* = 25) and CPJS group (*n* = 30). **(B)** The PCoA plots of microbial communities were based on operational taxonomic unit (OTU) composition. The placebo group (*n* = 25) and the CPJS group (*n* = 30) are respectively represented by different colors. **(C)** Differences in the gut microbiota between groups using linear discriminant analysis effect size (LEfSe) analysis at the genus level (*n* = 10). For taxa which were defined as unclassified, no rank, uncultured, or Incertae-Sedis, the name of the higher taxon level was added before its taxon abbreviation (g, genus; s, species). The symbol # indicates P<0.05.

After oral administration for 56 consecutive days, there was a subtle yet statistically significant difference in bacterial richness that emerged, as quantified by the ACE and Chao1 indices, indicating that CPJS is beneficial to the growth of gut microbiota. However, no significant differences in bacterial diversity were observed between the two groups, as assessed by the Shannon and Simpson indices (**Figure 5**), nor were there changes in the overall microbial structure (**Figure 5**). Some subtle beneficial changes in gut microbiota, which were associated with dietary consumption, were identified with the LEfSe analysis. As the threshold on the logarithmic LDA score was set at 3, a total of five genera were screened in the LEfSe analysis, including *Lactobacillus*, *Roseburia*, *Megasphaere*, *Phascolarctobacterium*, and *Weissellla* (**Figure 5**). These results indicated that long-term consumption of CPJS affects the bacterial abundance of gut microbiota in dyslipidemia.

## Discussion

This study revealed for the first time that CPJS is effective in improving energy metabolism, lipid metabolism, and amino acid metabolism and in enriching intestinal microbial flora. Consequently, it leads to the alleviation of dyslipidemia, manifested as decreased levels of TG, BMI index, and hip circumference over the course of treatment. These findings indicate that oral CPJS intervention could be beneficial and is non-harmful in the dyslipidemia population. Given the widespread availability of Chenpi and the public’s acceptance of natural products, recommending CPJS as an effective means of improving blood lipids in daily life is particularly appealing. As a novel functional food, Chenpi is utilized as raw materials and fermented alongside probiotics, ginseng, and other herbs, with the aim of addressing dyslipidemia. In our study, a significant decrease in TG level was observed in the treatment group after 4 consecutive weeks of oral CPJS administration. The underlying mechanism may be associated with the activation of TG-related hydrolysis pathways, including the peroxisome proliferator-activated receptor γ (PPARγ)-lipoprotein lipase (LPL)/adipose triglyceride hydrolase (ATGL), and farinol receptor (FXR)-hepatic esterase (HL) pathways, which collectively reduce the serum TG content. The primary component in CPJS that is responsible for lowering the lipid level is polymethyloxyl flavones, which are abundantly found in tangerine peel (27). *In vitro* experiments have demonstrated that polymethoxyl flavonoids can inhibit the activity of cholesterol regulatory element binding proteins (SREBPs) via the mTOR/p70s6k pathway, thereby exerting a lipid-lowering effect (28). Furthermore, the extract is capable of regulating the overall structure of intestinal flora, significantly enriching *Bacteroides ovatus* and modulating the levels of branched chain amino acids and aromatic amino acids, which contributes to the improvement of metabolic syndrome. Both edible fungal polysaccharides and crataegus flavonoids facilitate the balance of dyslipidemia by significantly reducing the TC, TG, and LDL-C levels while increasing HDL-C in hyperlipidemia rats, which is almost equivalent to simvastatin (29). The primary component responsible for the lipid-lowering effect of hawthorn is its flavonoids (30), and the mechanism underlying this effect involves the activation of PPARα and β-oxidation-related enzymes, the promotion of liver lipid degradation, and the subsequent reduction of blood lipid content (31). Additionally, the polysaccharide in *Lycium barbarum* has beneficial effects on lipid parameters such as TC, TG, and LDL-C, upregulating the level of Firmicutes, enhancing the diversity of gut microbiota, and mitigating metabolic disorders, ultimately alleviating weight gain in obese rats (32). These findings are consistent with our results. Notably, a significant decrease in BMI and hip circumference has been observed following the oral administration of CPJS in the treatment group.

Dyslipidemia arises as a comprehensive consequence of multiple metabolic pathway abnormalities and function disorders, encompassing lipid metabolism, amino acid metabolism, energy metabolism, and other perturbations. In our study, we observed the enrichment of multiple lipid metabolic pathways in the serum of the treatment group, mainly including glycerophospholipid metabolism, linoleic acid metabolism, arachidonic acid metabolism, and phosphatidylinositol signaling system, among others.

Phosphatidylcholine (PC) is the primary membrane-forming phospholipid in eukaryotes and serves as a ligand for specific receptors, playing a crucial role in signal transduction (33). It is essential for lipoprotein assembly and secretion and plays a significant role in regulating cellular lipid metabolism and lipid homeostasis (34). Lysophosphatidylcholine (lysoPC), a metabolite of PC produced through phospholipase metabolism, binds to its receptor G protein coupled receptor (G2A) to activate MAPK pathway, including ERK1/2, p38MAPK, JNK, and Toll-like receptors. This activation leads to the induction of oxidative stress, chemokine expression, and inflammatory response (35). Studies have shown that LysoPC levels are elevated in obese individuals compared to healthy individuals, and LysoPC is increasingly recognized as a key marker positively associated with cardiovascular and neurodegenerative diseases (36, 37). Additionally, trimethylamine N-oxide (TMAO), synthesized by gut microbiota through the metabolic conversion of choline and phosphatidylcholine, is a known atherogenic factor that can prevent myocardial infarction and post-myocardial infarction ventricular remodeling (16). The serum levels of the PC metabolite have been positively correlated with the risk of CVD (38). In this study, we observed that multiple PCs were regulated in the serum of patients in the treatment group, with significant decreases in LysoPC (18:0) and LysoPC (20:2) levels. These findings suggested that CPJS can regulate the levels of PC and LysoPC through the glycerophospholipid metabolism, arachidonic acid metabolism, linoleic acid metabolism, and α-linolenic acid metabolism pathways, thereby modulating biological processes such as lipid metabolism, oxidative stress, and inflammatory response in patients with dyslipidemia

Several studies have focused on the metabolic mechanism of underlying dyslipidemia, highlighting amino acids as crucial endogenous signaling molecules of lipid metabolism (39, 40). β-alanine, a non-essential amino acid in humans, plays a vital role in cell metabolism by providing energy, regulating acid–base balance, and modulating protein synthesis. In the context of disease, β-alanine metabolism can be affected, with studies reporting abnormal β-alanine metabolism in individuals with dyslipidemia (41). In our study, we observed a decrease in β-alanyl-arginine content in the treatment group, suggesting a relationship between β-alanyl-arginine and β-alanine. This finding implies that CPJS intake may be involved in regulating β-alanine metabolism, thereby modulating energy and lipid metabolism. Tryptophan is an essential amino acid which is involved in various physiological processes, including neuronal function, immunity, and intestinal homeostasis. The kynurenine pathway is a key metabolic route for the degradation of approximately 95% of free tryptophan, primarily initiated by two crucial rate-limiting enzymes: indoleamine-2,3-dioxygenase (IDO) and tryptophan2,3-dioxygenase (TDO) (42). Under physiological conditions, TDO, induced by the activation of the hypothalamic–pituitary–adrenal (HPA) axis with increased glucocorticoid levels, catalyzes the degradation of 90% of tryptophan in the liver and brain (43). In our study, although no reduction in kynurenine content was observed, the tryptophan content decreased in the treatment group after the intervention. This suggests that CPJS may be involved in the regulation of the tryptophan metabolism pathway, although the specific mechanism remains to be elucidated.

As a hub of neuro-endocrine-immunoregulatory network, the HPA axis is crucial for maintaining the internal environment balance in the body, and its normal activation is necessary for the homeostasis of lipid and glucose (44). When an individual experiences psychological or physical stress, the HPA axis responds by increasing cortisol secretion. Long-term unalleviated stress can result in chronic overactivation of the HPA axis and persistent release of glucocorticoids, and this gradually leads to the gradual accumulation of visceral fat and a range of overlapping conditions, including central obesity, hypertension, dyslipidemia, and disrupted glucose metabolism. Additionally, chronic inflammation, adipokine dysregulation, and oxidative stress may also develop as pathophysiological sequelae (45). Glucocorticoids are known to alter hepatic lipid metabolism and exacerbate dyslipidemia induced by an unhealthy diet (46). In this study, we observed a significant decrease in serum cortisol levels in the treatment group, suggesting that CPJS may regulate the steroid hormone biosynthesis pathway, thereby modulating blood lipids and improving dyslipidemia.

Jiaosu has garnered significant attention in recent years for their potential to regulate human intestinal microorganisms. Numerous studies have demonstrated that these enzymes can enhance the composition of the intestinal flora. In our study, we found that CPJS has the ability to enhance the abundance of specific intestinal microorganisms, including *Lactobacillus*, *Roseburia*, *Megasphaera*, *Phascolarctobacterium*, and *Weissella*, representing one of the potential underlying mechanisms for regulating lipid metabolism and optimizing lipid profiles. *Lactobacillus* belongs to a genus of gram-positive, aerotolerant anaerobes or microaerophilic, rod-shaped, non-spore-forming bacteria (47). The primary functions of *Lactobacillus* include maintaining flora balance, lowering cholesterol levels, enhancing immunity, and promoting nutrient absorption (48). A novel polysaccharide, GPH1, has been found to significantly reduce body weight, alleviate liver lipid accumulation, and mitigate inflammatory damage by promoting the proliferation of *Lactobacillus* and other intestinal bacteria (49). Studies have reported that hawthorn leaf flavonoids significantly regulate the relative abundance of major bacteria, including *Lactobacillus* (50). In a study investigating the associations between gut microbiota features and serum lipid profiles based on sex differences in a Chinese population, it was observed that that *Roseburia* abundance was positively correlated with serum TG level but was decreased in male patients with low high-density lipoprotein cholesterol compared to the controls (51). Karlsson et al. noted an enrichment of *Roseburia* in CVD patients, while a lower abundance of *Roseburia* was observed in patients with diabetes (52). Previous studies have demonstrated that *Megasphaera* is enriched in healthy controls in a previous study (53), which aligns with our findings. Specifically, we observed a notable increase in the abundance of *Megasphaera* after oral administration of CPJS. However, conflicting results were reported in another study investigating gut microbiota characteristics in patients with hypertension and/or dyslipidemia (54). The *Weissella* genus belongs to the phylum Firmicutes and the family Lactobacillaceaae. *W. cibaria* has been shown to exhibit anti-obesity effects by regulating lipid metabolism in mice with high-fat-diet-induced obesity (55). Short-chain fatty acids (SCFAs), produced during the fermentation process of gut microbiota utilizing undigested dietary fiber as energy source, play a complex role in lipid metabolism. In particular, propionate and acetate induce satiety and inhibit cholesterol synthesis (56). *Weissella* is associated with SCFA production, especially propionate (57).

Our study revealed that CPJS exhibited favorable safety and tolerability in the management of dyslipidemia, offering robust evidence to support its application as a supplementary approach to lipid reduction. Nonetheless, certain limitations were identified. The small sample size may introduce population bias into the findings, and the lack of subgroup analysis among participants could obscure some genuinely representative results. To fully leverage the considerable potential of Chenpi Jiaosu in regulating blood lipids and facilitate its clinical adoption, future research should aim to expand the sample size, validate its effects across diverse subgroups of individuals with dyslipidemia, and delve deeper into its specific mechanisms of action.

## Conclusions

The study demonstrates the efficacy and safety of CPJS administration in regulating lipid level in patients with dyslipidemia. The results indicate that TG level and BMI were significantly improved in the treatment group. Based on metabolomics analysis and gut microbiota investigation, the potential underlying mechanisms may involve enhancements in energy metabolism, lipid metabolism, amino acid metabolism, and an increase in specific intestinal microorganisms.

## Data Availability

Publicly available datasets were analyzed in this study. This data can be found in the NCBI SRA database with the following identifiers: Accession URL: https://www.ncbi.nlm.nih.gov/sra/PRJNA1234548 Project ID: PRJNA1234548 (SRA ID: 1234548).

## Supplementary material

Supplementary Figure 1 Measure scores of primary outcomes with improving trend. TG, Triglyceride; TC, Total cholesterol; HDL-C, High-Density Lipoprotein Cholesterol; LDL-C, Low-Density Lipoprotein Cholesterol.

Supplementary Table 1 Secondary outcome measure scores.

Supplementary Table 2 Identification of metabolites in serum samples after the oral administration of CPJS.

Supplementary Table 3 Identification of the metabolic pathways in serum.
