# Supplementary figures and images for: Effects of Chenpi Jiaosu on serum metabolites and intestinal microflora in a dyslipidemia population: a randomized controlled pilot trial
Source: Front Endocrinol (Lausanne). 2025 Mar 28;16:1552117. doi: 10.3389/fendo.2025.1552117 (PMC11985429; doi:10.3389/fendo.2025.1552117)

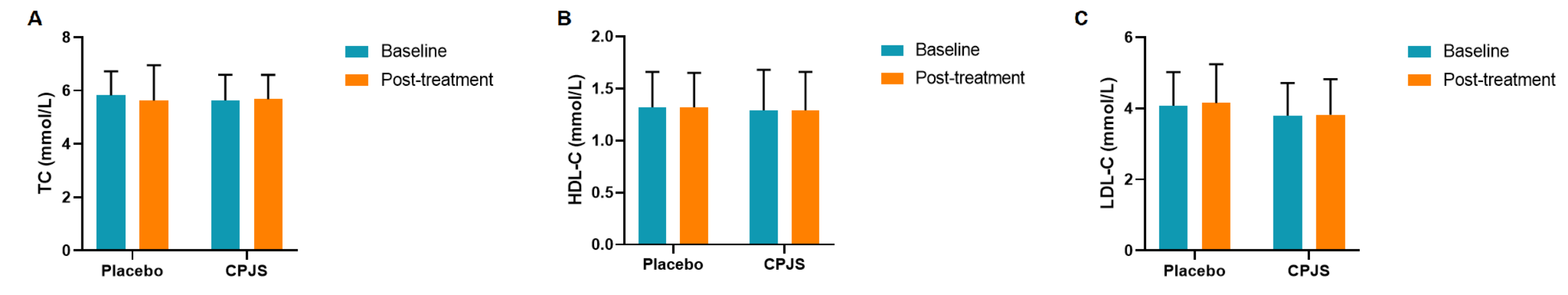

Supplement: Supplementary Figure 1 — Measure scores of primary outcomes with improving trend. TG, Triglyceride; TC, Total cholesterol; HDL-C, High-Density Lipoprotein Cholesterol; LDL-C, Low-Density Lipoprotein Cholesterol. [file Image1.tif]

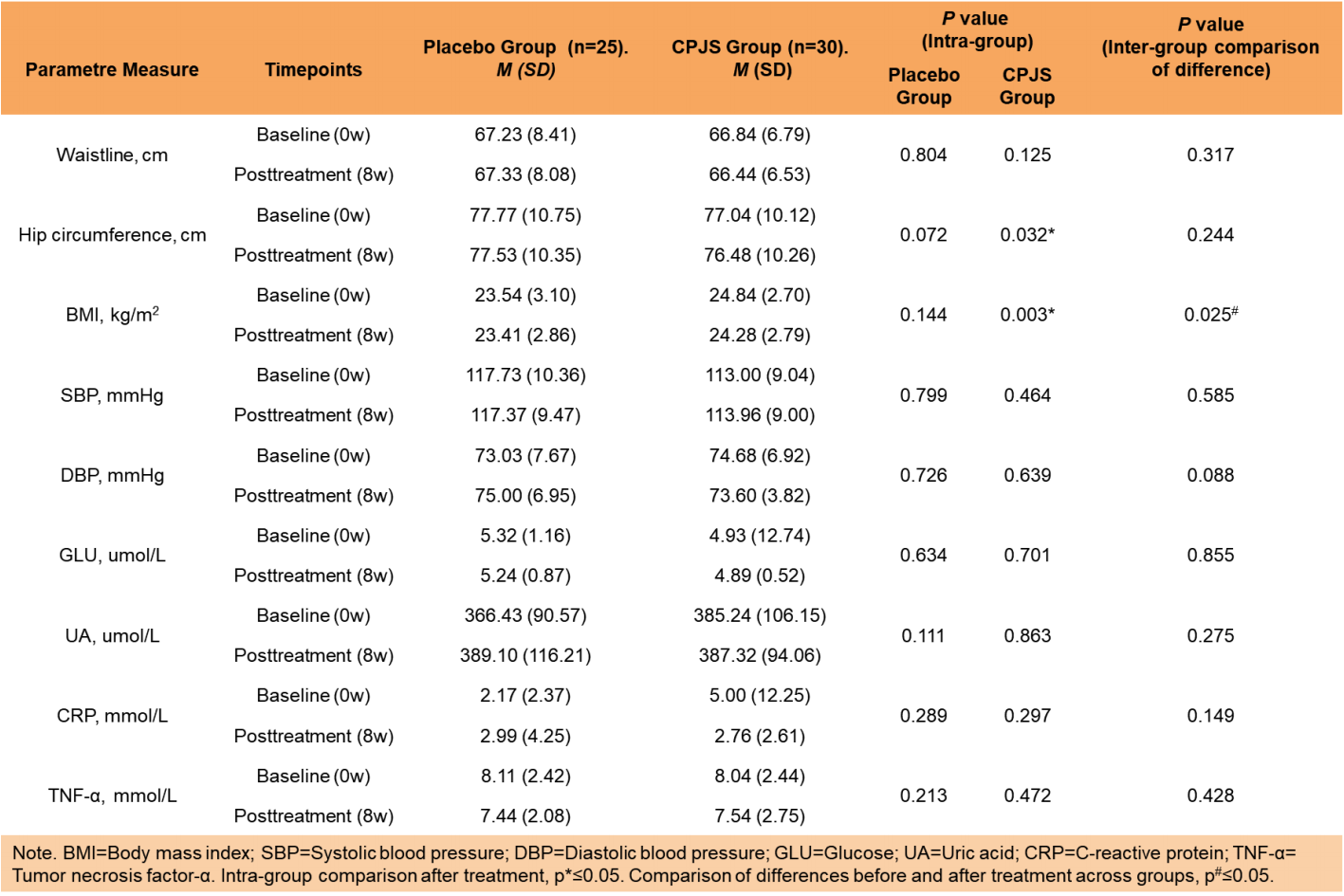

Supplement: Supplementary file 2 [file Image2.tif]

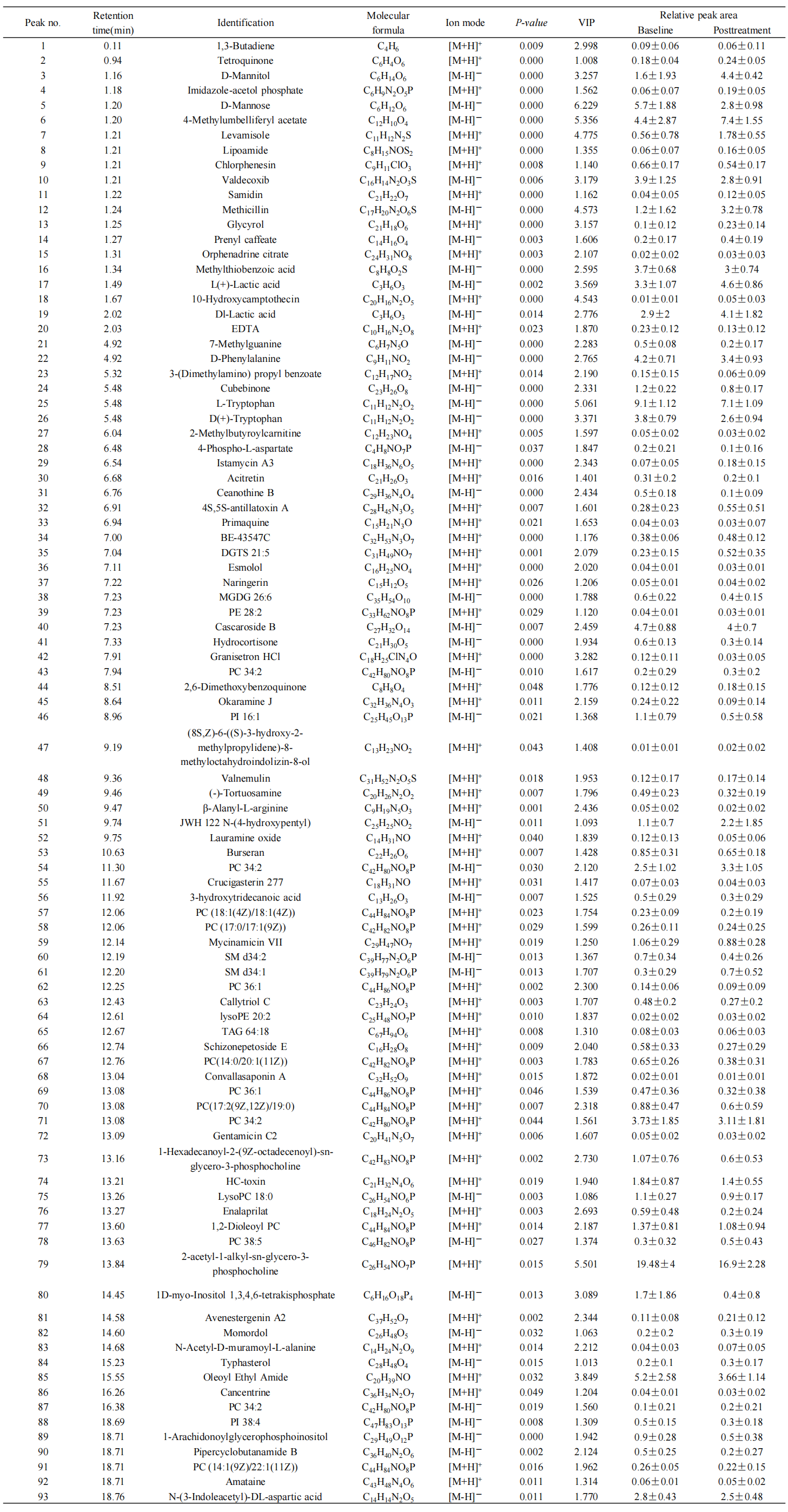

Supplement: Supplementary file 3 [file Image3.tif]

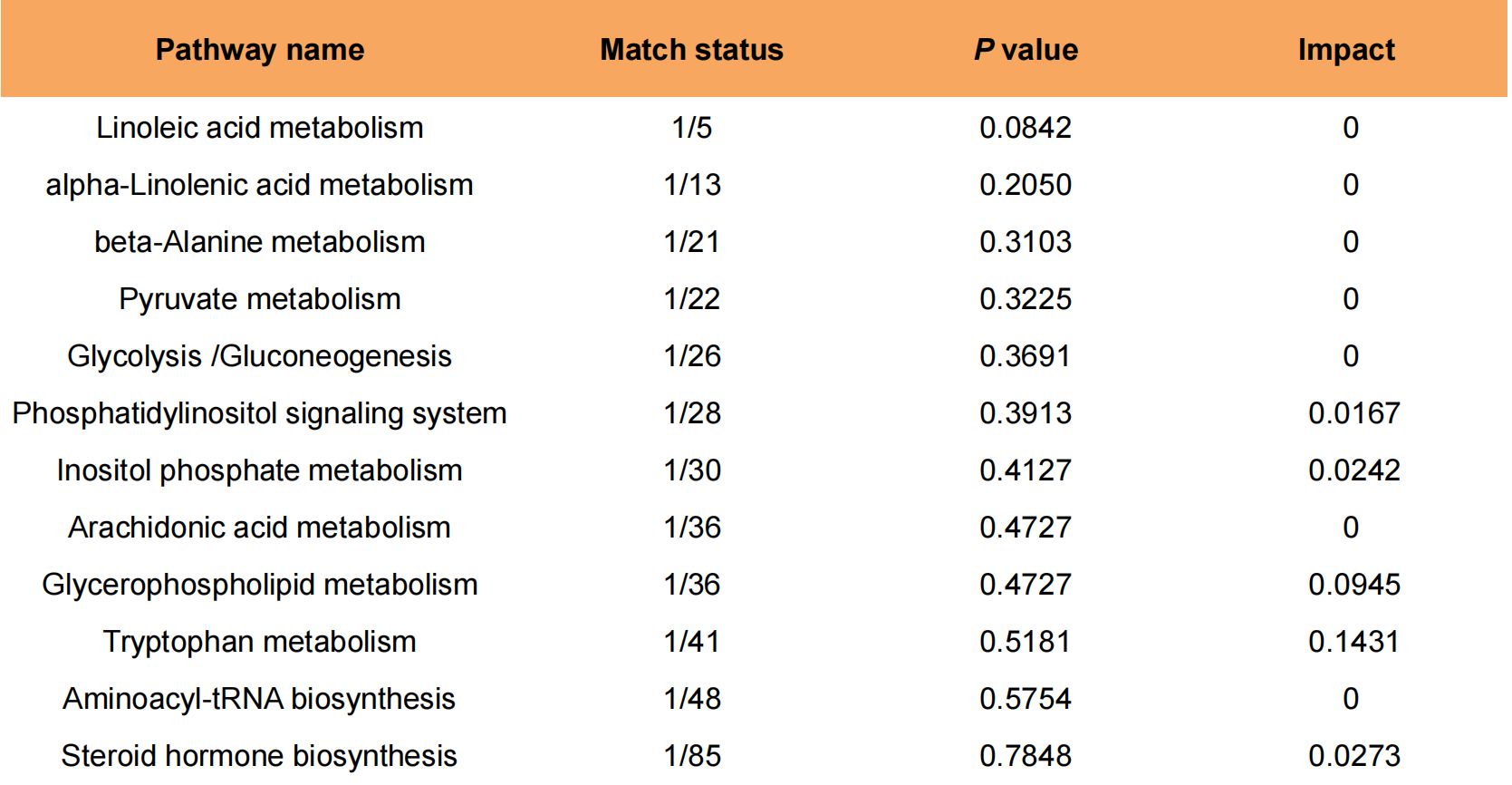

Supplement: Supplementary file 4 [file Image4.tif]
