# Supplementary material for: Effects of Chenpi Jiaosu on serum metabolites and intestinal microflora in a dyslipidemia population: a randomized controlled pilot trial
Source: Front Endocrinol (Lausanne). 2025 Mar 28;16:1552117. doi: 10.3389/fendo.2025.1552117 (PMC11985429; doi:10.3389/fendo.2025.1552117)
Supplement: Supplementary Table 1 — Secondary outcome measure scores. [file DataSheet1.pdf]

Supplementary 1. Quality Control Standard of Chenpi Jiaosu

---

1. Scope

This document specifies the test methods, inspection rules, labeling, packaging, transportation, and storage requirements for concentrated beverages. It applies to the production, inspection, and sale of Xinhui Chenpi Jiaosu concentrate beverage produced by Xinbaotang.

---

2. Normative References

The following documents are essential for the application of this standard. For dated references, only the cited edition applies. For undated references, the latest version (including all amendments) applies.

- **GB/T 191**: Packaging, storage, and transportation symbols
- **GB 2762**: National food safety standards – Limits of pollutants in food
- **GB 4789.1**: National food safety standards – General rules for microbiological examination of food
- **GB 4789.2**: National food safety standards – Determination of total bacterial count
- **GB 4789.3**: National food safety standards – Determination of coliform bacteria
- **GB 4789.4**: National food safety standards – Salmonella testing
- **GB 4789.10**: National food safety standards – Staphylococcus aureus testing
- **GB 4789.15**: National food safety standards – Mold and yeast count testing
- **GB 4789.21**: Microbiological examination of frozen beverages and drinks
- **GB 4789.26**: National food safety standards – Commercial sterility testing
- **GB 4789.35**: National food safety standards – Lactic acid bacteria testing
- **GB 5009.11**: National food safety standards – Determination of total arsenic and inorganic arsenic in food
- **GB 5009.12**: National food safety standards – Determination of lead in food
- **GB 5009.15**: National food safety standards – Determination of cadmium in food
- **GB 5009.17**: National food safety standards – Determination of total mercury and organic mercury in food
- **GB 5009.44**: National food safety standards – Determination of sodium chloride in food
- **GB 5009.123**: National food safety standards – Determination of chromium in food
- **GB 5009.157**: National food safety standards – Determination of organic acids in food
- **GB 7718**: National food safety standards – General rules for prepackaged food labeling
- **GB/T 10468**: Methods for determining the pH of fruit and vegetable products
- **GB/T 12143**: General analytical methods for beverages
- **GB 12456**: National food safety standards – Determination of total acid in food
- **GB 14881**: National food safety standards – General hygiene requirements for food production
- **GB 28050**: National food safety standards – General rules for nutrition labeling of prepackaged foods
- **GB/T 18672**: Wolfberry
- **GB/T 30383**: Ginger
- **DB4407/T 69**: Geographical indication product – Xinhui orange
- **DB4407/T 70**: Geographical indication product – Xinhui tangerine peel

- **DBS 22/024**: Local food safety standards – Ginseng as a food raw material
- **GH/T 1159**: Hawthorn
- **T/CNHFA 111.6**: Raw materials for health food – Poria cocos
- **QB/T 4587**: γ-Aminobutyric acid
- **SN/T 4260**: Determination of crude polysaccharides in exported plant-derived foods by the phenol-sulfuric acid method
- **T/CBFIA 08001**: Jiaosu product classification guidelines
- **T/CBFIA 08002**: Good production practices for food enzymes
- **The Pharmacopoeia of the People's Republic of China**
- **Measures for Metrological Supervision and Administration of Quantitative Packaging Commodities** (Order of General Administration of Quality Supervision, Inspection, and Quarantine [2005] No. 75)

---

3. Terms and Definitions

For the purposes of this document, the terms and definitions in **T/CBFIA 08001** apply. Additionally:

- **Chenpi Jiaosu**: A fermented product containing specific bioactive ingredients, produced through the fermentation of Xinhui orange pulp, barbary wolfberry, ginseng, hawthorn, ginger, and Poria cocos by lactic acid bacteria and yeast.

---

4. Quality Requirements

4.1 Requirements for Raw Materials and Auxiliary Materials

- **Xinhui orange**: Complies with **DB4407/T 69** (Geographical indication product – Xinhui orange).
- **Xinhui tangerine peel**: Complies with **DB4407/T 70** (Geographical indication product – Xinhui tangerine peel).
- **Barbary wolfberry**: Complies with **GB/T 18672** (Wolfberry).
- **Ginseng**: Complies with **DBS 22/024** (Local food safety standards – Ginseng as a food raw material).
- **Hawthorn**: Complies with **GH/T 1159** (Hawthorn).
- **Ginger**: Complies with **GB/T 30383** (Ginger).
- **Poria cocos**: Complies with **T/CNHFA 111.6** (Raw materials for health food – Poria cocos).

4.2 Sensory Requirements

Complies with the provisions of **Table 1**.

Table 1: Sensory Requirements

Parameter	Requirement	Inspection Method
**Color and luster**	Black liquid	Randomly select 50 mL of a well-mixed sample in a dry, clean cup under indirect light. Visually observe for appearance, color, and foreign matter.
**Impurities**	No visible foreign impurities	Visual inspection under normal lighting conditions.
**Taste and odor**	No abnormal odor	Gently smell the sample to assess aroma. Taste a small amount using the tip of the tongue.

4.3 Physicochemical Indicators

- **General physicochemical indicators**: Comply with **Table 2**.
- **Characteristic physicochemical indicators**: Comply with **Table 3**.

Table 2. General Physicochemical Indicators

Parameter	Indicator	Inspection Method
**pH**	≤ 4	**GB/T 10468**
**Ethanol content (g/100g)**	≤ 1.5	**GB/T 12143**
**Sodium chloride (g/100g)**	≤ 0.2	**GB 5009.44**

Table 3. Characteristic Physicochemical Indicators

Parameter	Indicator	Inspection Method
**Total acid (as lactic acid, g/100g)**	18	**GB 12456**
**Crude polysaccharides (g/100g)**	3	**SN/T 4260**
**γ-Aminobutyric acid (mg/kg)**	≥ 120	**QB/T 4587**

4.4 Ginseng Thin-Layer Chromatography Identification

1. **Sample Preparation**:
   - Take 30 mL of the product, extract with n-butanol (3 × 30 mL), combine the n-butanol layers, and wash with 2% sodium hydroxide solution (3 × 30 mL).
   - Evaporate the n-butanol solution, dissolve the residue in 1 mL of methanol, and use as the test solution.
2. **Control Preparation**:
   - Weigh 1 g of ginseng reference material, reflux with 40 mL of chloroform for 1 hour, discard the chloroform, dry the residue, and dissolve in 1 mL of water-saturated n-butanol.
3. **Thin-Layer Chromatography**:
   - Apply 10 μL and 5 μL of the test and control solutions to a silica gel G plate.
   - Develop in a pre-saturated chamber with chloroform-ethyl acetate-methanol-water (15:40:22:10).
   - Dry the plate, spray with 10% sulfuric acid in ethanol, and heat at 105°C until spots appear.
   - Observe under UV light (365 nm). The test solution should show fluorescent spots at Rf 0.6 and Rf 0.5, matching the control.

4.5 Food Safety Indicators

4.5.1 Pollutant Limits

Comply with **GB 2762**.

4.5.2 Microbiological Limits

- **Commercial sterile products**: Comply with commercial sterility requirements.
- **Non-commercial sterile products**: Comply with **Table 5**.

Table 5. Microbiological Limits

Parameter	Sampling Plan (n, c)	Limit (CFU/g or mL)	Inspection Method
**Total bacterial count**	5, 2	m = 10^2, M = 10^3	**GB 4789.2**
**Coliform bacteria**	5, 2	m = 1, M = 10	**GB 4789.3**
**Staphylococcus aureus**	5, 1	m = 10^2, M = 10^3	**GB 4789.10**
**Salmonella**	5, 0	0	**GB 4789.4**
**Mold**	5	≤ 20	**GB 4789.15**

4.6 Production Specifications

Comply with **GB 14881** and **T/CBFIA 08002**.

---

5. Inspection Rules

5.1 Batch Inspection

Products shall undergo batch-by-batch inspection by the manufacturer's quality control department. Only qualified products, accompanied by a quality certificate, shall be released.

5.2 Batch Definition

A batch consists of products of the same formulation, specification, and production run.

5.3 Sampling

Randomly select samples from finished products. The total sample size shall be ≥ 2 kg (or ≥ 2000 mL for liquids).

5.4 Factory Inspection

- **Commercial sterile products**: Inspect sensory properties, pH, ethanol content, sodium chloride, total bacterial count, mold, and coliform bacteria.
- **Non-commercial sterile products**: Inspect sensory properties, pH, ethanol content, sodium chloride, and coliform bacteria.

5.5 Type Testing

- Conduct annually or under specific conditions (e.g., new product trials, raw material changes, or regulatory requests).
- Test items include those specified in Sections 5.2–5.4 and at least three indicators from **Table 3**.

5.6 Judgment Rules

- If all samples pass, the batch is qualified.
- If sensory or physicochemical indicators fail, double the sample size and retest. The retest result is final.
- If any food safety indicator fails, the batch is disqualified without retesting.

---

6. Labeling, Packaging, Transportation, and Storage

6.1 Labeling

Prepackaged product labels shall comply with **GB 7718** and **GB 28050**, including:

- Product name, ingredient list, nutrition label, net content, manufacturer's name and address, production date, shelf life, storage conditions, food production license number, and product standard number.

6.2 Packaging

Packaging shall be intact and comply with relevant national or industry standards.

6.3 Transportation

Avoid sunlight, rain, and heavy pressure. Do not transport with toxic, odorous, volatile, corrosive, or radioactive materials.

6.4 Storage

Store in a clean, dry, ventilated area away from pests and harmful substances. Do not store with odorous materials.
